# Food protein-induced enterocolitis syndrome in a tertiary pediatric center: safety of guideline-conforming food challenges

**DOI:** 10.1186/s13223-022-00694-y

**Published:** 2022-06-16

**Authors:** Samantha Wong, Lucy Duan, Alana Galper, Adelle Atkinson, Julia Upton, Thomas Eiwegger

**Affiliations:** 1 Division of Immunology and Allergy, Department of Paediatrics, The Hospital for Sick Children, University of Toronto, Toronto, Canada; 2 Translational Medicine Program, Research Institute, The Hospital for Sick Children, Toronto, Canada; 3 Department of Immunology, University of Toronto, Toronto, Canada; 4 Department of Pediatric and Adolescent Medicine, University Hospital St. Pölten, Dunant-Platz 1, 3100 St. Pölten, Austria; 5 Karl Landsteiner University of Health Sciences, Krems, Austria

**Keywords:** FPIES, Food allergy, Oral food challenges, Non-IgE allergy

## Abstract

Food protein-induced enterocolitis syndrome is a non-IgE-mediated reaction to food that is poorly understood, and underdiagnosed. Trigger foods can belong to any food group, but are most commonly milk, soy, rice, oat, egg, and fish. In this retrospective study (2015–2020), we describe the clinical presentations and triggers of 37 children referred to tertiary hospital with a confirmed or suspected diagnosis of food protein-inducted enterocolitis. We reviewed the safety of current recommendations by looking at the outcome of 24 oral food challenges. All of these patients presented with clear cut systemic reactions including lethargy. We also assessed the severity of the reactions. Oral food challenges occurred in the hospital day unit with the majority of patients having IV access in place. Despite a clear history of FPIES with lethargy and the requirement for re-hydration of the challenged population, 21/24 (88%) of the FPIES OFCs were successful. Of the three patients who reacted, symptoms were of moderate nature, mainly vomiting. This highlights the importance of early diagnosis and a pro-active approach to performing guideline-directed oral food challenges in patients with food protein-induced enterocolitis syndrome.

## Main text

Food protein-induced enterocolitis syndrome (FPIES) is a non-IgE-mediated reaction to food that is poorly understood, and underdiagnosed [1]. Reactions typically present during infancy between 2 and 12 months of age [1–7]. Trigger foods can belong to any food group, but are most commonly milk, soy, rice, oat, egg, and fish [1–7]. Diagnosis of FPIES is based on clinical symptoms as there are no reliable diagnostic serum markers available at this time [7]. There is emerging evidence that an increase of neutrophils, platelets and some serological parameters could be helpful [7].

Acute FPIES reactions typically manifest 1–4 h after ingesting the trigger food, and include delayed protracted vomiting, lethargy, pallor, and diarrhea which can lead to hypotension and cardiovascular shock [1, 2, 4, 5, 7]. Chronic FPIES presents differently with poor weight gain and failure to thrive (FTT), and may take a longer time to recognize and diagnose [1, 7]. Most patients present with FPIES to a single food trigger, but 10–35% patients can have multiple trigger foods [3, 5, 7]. Resolution of FPIES is a common phenomenon and may vary by type of food trigger and geographical location. Some cases can persist throughout childhood, and there are also reports of FPIES in adults [2, 5, 8]. Concomitant allergic sensitization to the same food can occur (5–30%) [3]. These patients with atypical FPIES may have a more protracted course or can transform into a more classical IgE-mediated allergy [3, 6]. Data on the predominance of triggers and expected resolution of FPIES in Canada is limited.

In this retrospective study (2015–2020), we describe the clinical presentations, triggers, and outcomes of oral food challenges (OFCs) of 37 children referred with a confirmed or suspected diagnosis of FPIES to our tertiary pediatric hospital. FPIES was diagnosed and managed by allergists using the International Consensus Guidelines for the Diagnosis and Management of Food Protein-Induced Enterocolitis Syndrome [1]. FTT was documented explicitly in clinic notes or growth charts. The study was approved by the research ethics board at the Hospital for Sick Children (REB#1000071021).

Thirty-seven patients were diagnosed with acute FPIES (Table 1). Referral was made for clinical evaluation pre-OFC (11/37) or for a second opinion for suspected FPIES. Seventeen participants had more than one suspected trigger food (46%). There were 71 reported food triggers in 37 patients. The most common foods were oat, milk, egg, and rice. Soy, a common trigger in US populations was reported in 4/37 of our patients [2]. All 37 patients had a history of protracted vomiting; 16/37 reported lethargy, 15/37 reported diarrhea, 11/37 experienced pallor, and 4/37 had loss of consciousness. One patient had documented hypotension and dizziness. The onset of symptoms occurred on average 2–3 h (123 ± 64 min) after ingestion of the culprit food, which is consistent with prior descriptions that the majority of OFC reactions occur around 121 min [6]. In keeping with existing literature, the average age at first exposure to suspected trigger foods was 9.6 months [1–7]. Six participants reported difficulty/hesitation introducing new foods after provision of a food introduction guide and required additional dietary counselling.

**Table 1:** Patient characteristics

	N/value (%)
Gender	
Male	23 (62.2)
Female	14 (37.8)
Age (years)	
Mean (SD)	3.2 (3.2)
Min	0.5
Max	17
Atopy	
Eczema, yes	14 (37.8)
Eczema, no	22 (59.5)
Asthma, yes	11 (29.7)
Asthma, no	25 (67.6)
History of FTT	
Yes	4 (10.8)
No	22(59.5)
Unknown	11 (29.7)
FPIES type	
Acute	34 (98.9)
Chronic	2 (5.4)
Unclear	1 (2.7)
Multiple suspected trigger foods	
Yes	17 (45.9)
No	20 (54.1)
History of FPIES challenge prior to clinic visit	
Yes	8 (21.6)
No	29 (78.4)
Oral food challenges for FPIES	
Yes	24 (56.8)
Patients with positive SPT or sIgE	1/24
No	13 (24.3)
Patients with positive SPT or sIgE	1/13
Pending challenge (scheduled)	7 (66.7)
Home introduction successful	2 (5.4)
Home introduction failed	1 (2.7)
Appointment cancelled	3 (5.4)
Food challenged	
Cow’s Milk	6 (28.6)
Oat	5 (23.8)
Egg (stove-top cooked)	2 (9.5)
Egg (baked egg muffin)	1 (4.8)
Soy	2 (9.5)
Rice	1 (4.8)
Chicken	1 (4.8)
Beef	1 (4.8)
Wheat	1 (4.8)
Goat meat	1 (4.8)

Ten patients had eczema and seven had asthma alone. Four patients had both asthma and eczema, Sensitization to the trigger food [skin prick test (SPT) or IgE-positivity] was reported in 2/37 (5.4%) patients. One patient had a positive SPT to egg and underwent a modified protocol in the form of a graded food challenge. Incremental doses were given every 15–20 min and stopped at the cumulative dose requested for FPIES, with success [2]. A second with protracted vomiting 3 h after egg ingestion suggestive of an FPIES reaction to egg with elevated specific IgE to egg white and is scheduled for an OFC.

Twenty-seven of the 37 children were reintroduced to the suspected food, 24 by medically supervised OFC, 2 by home introduction based on low-risk history (more than 2 years from last reaction, isolated vomiting with initial reaction, no other interventions required). One patient refused to eat the suspected food in clinic which was attributed to the setting. The subsequent attempt to perform a home introduction resulted in protracted vomiting and an emergency department visit. The remaining patients are waiting for their OFCs, cancelled appointments due to illness or—more recently—due to the COVID-19 pandemic.

Of the patients who underwent OFCs, the average age at OFC was 4 years. The median time since patients’ last exposure to the food was 24 months. This is in the upper range of the 12–24 months recommended before considering OFC and reflects the, often prolonged, time to diagnosis [1, 3]. SPTs of the allergens to be challenged with were performed the day of OFC. Despite a clear history of FPIES with lethargy and the requirement for re-hydration, 21/24 (88%) of the FPIES OFCs were successful. This highlights the importance of following the guideline-recommended interval of waiting 12–24 months from the last reaction before OFC.

The majority of OFCs (n = 18) were performed with IV access in place. Patients with a milder course of reaction upon the last recalled exposure without lethargy received an OFC in the outpatient ward without IV access. OFC dosing was in accordance with the International Consensus Guidelines: 0.06–0.60 g food protein /per kg body weight not exceeding 3 g food protein, or 10 g of food divided into three equal doses given over 30 min. Patients were observed for a minimum of 4 h [2].

Two out of three children who had a positive FPIES OFCs were 3 years or older and all had protracted vomiting. One patient received IV ondansetron (0.15 mg/kg body weight), and 2 received sublingual ondansetron. One reaction occurred to egg, and the patient required IV fluids and hospital admission overnight. The second and third reactions occurred to soy. In one case the reaction was self-limiting, and no IV fluids were required. The third patient, improved after administration of IV fluids and ondansetron, and was discharged the same day. Given the significant resources needed for challenge procedure with an IV access in place, additional studies on the use of oral hydration, and PO/IM ondansetron would be helpful to determine more accurately whether more OFCs could be performed at home or in an outpatient instead of a daycare clinic setting.

In conclusion, we report on 37 children at a tertiary pediatric center diagnosed with FPIES, and the outcome of 24 OFCs. 21/24 had successful OFCs, which highlights the importance of early diagnosis and a pro-active approach to performing guideline-directed OFCs in patients with FPIES.

## Data Availability

The datasets generated and/or analysed during the current study are not publicly available due to the fact that this is patient data and if made available may lead to a breach of privacy. The datasets are available from the corresponding author on reasonable request.

## Abbreviations

SickKids: The hospital for sick children
FPIES: Food protein-induced enterocolitis syndrome
OFC: Oral food challenge
SPT: Skin prick test
IgE: Immunoglobulin E
